# Hyper-Cross-linked Cellulose Nanofibrils with Spontaneous and Reversible Adsorption of Aromatic Pollutants from Water as a Valid Alternative to Fossil-Based Adsorbents

**DOI:** 10.1021/acsami.5c05009

**Published:** 2025-06-20

**Authors:** Antonio Maglione, Federico Olivieri, Roberto Avolio, Rachele Castaldo, Mariacristina Cocca, Maria Emanuela Errico, Veronica Ambrogi, Gennaro Gentile

**Affiliations:** † Institute for Polymers Composites and Biomaterials, National Research Council of Italy, Via Campi Flegrei 34, 80078 Pozzuoli, Italy; ‡ Department of Chemical, Materials and Industrial Production Engineering, University of Naples Federico II, P.le Tecchio 80, 80125 Napoli, Italy

**Keywords:** cellulose, hyper-cross-linking, adsorption, regeneration, aromatic pollutants

## Abstract

In this work, a novel high surface area adsorbent based on cellulose and inspired by hyper-cross-linked polymers was designed. Cellulose nanofibrils (CNF) were functionalized with poly­(vinylbenzyl chloride) and hyper-cross-linked through Friedel–Crafts alkylation, yielding a micro/mesoporous material characterized by a specific surface area of 409 m^2^/g, microporous fraction of 50%, and biobased content of about 70 wt %. The functionalized CNF, tested for the adsorption of 2,4-dichlorophenol (DCP) from water at 298 K, were able to remove 90% of the pollutant from a 62.5 mg/L DCP solution and adsorb 284 mg/g at a higher concentration (1000 mg/L). Thermodynamic studies demonstrated the multilayer adsorption of the hyper-cross-linked CNF, the exothermic nature of the process, and its spontaneity. The hyper-cross-linked cellulose nanofibrils were reusable with efficiency above 98% in 5 subsequent cycles. The adsorption performance was stable across varying pH levels, and interference from natural organic matter (e.g., humic acids) was minimal (<10%). This work marked a promising step toward more sustainable sorbent materials by demonstrating the potential of cellulose nanofibrils as functional scaffolds. The strategy could be extended to waste-derived cellulose sources and biobased aromatic compounds, paving the way for fully renewable porous adsorbents.

## Graphical abstract

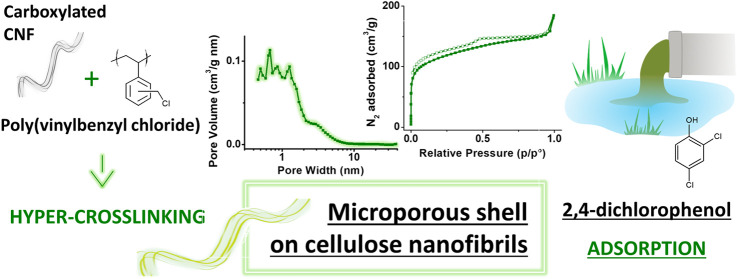

## 1 Introduction

Water pollution is a critical problem that affects a large part of the world. Both developed and developing countries are facing this issue. Water quality is influenced by many factors like climate, geology, and, in particular, human activities. The greatest threat to water quality is posed in large part by point sources of industries, municipalities, and agriculture.^1^ Above all, the great development of printing, oil refining, electroplating, mining, and chemical industries has made water pollution a serious issue.^2,3^ Organic pollutants like dyes, antibiotics, phenols, or heavy metal pollutants are only a few classes of materials that have been found in water reserves.^4^ Chlorophenols, notably, are produced as effluents of several industrial and agriculture companies, thus leading to accumulation and contamination in the ecosystem and food chain,^5^ and they affect human health even at low concentrations.^6^ They are raw materials used for herbicides, pharmaceutical intermediates, wood preservatives, and pesticides.^7^ They are resistant to biodegradation and are therefore persistent in the environment. Due to their high toxicity, potential carcinogenicity, and corrosive capacity, they are considered as priority pollutants by the Environmental Protection Agency (EPA).^3^

As reported by Fan et al.,^6^ many methods have been developed to treat contaminated water in purification processes; among them, adsorption is considered a very interesting choice. Physical adsorption, in particular, is a simple, versatile, cost-effective, and environmental-friendly process due to the absence of toxic byproducts, its spontaneity, and its reversibility, which implies the possibility to regenerate the adsorbents.^8^

High specific surface area (SSA) materials show excellent properties in adsorption processes.^9^ Several porous materials can be used as adsorbents, both natural and manmade: clay,^10^ zeolites,^11,12^ activated carbons,^13^ and microporous organic polymers (MOP).^14,15^ Among these, hyper-cross-linked resins (HCLRs) are receiving growing attention because of their advantages, such as various preparation methods, easy functionalization, large specific surface area (SSA), cheap reagents, and mild reaction conditions.^16^ HCLRs are typically characterized by high SSA up to about 2000 m^2^/g, porosity ranging prevalently from micropores to small mesopores, and high chemical and thermal resistance.^17–19^ In addition, the porosity and functionality of HCLR can be tailored in many ways to achieve different results^18^ and enlarge their applications, such as imparting magnetic properties^19^ or enhancing the adsorption of specific pollutants, such as CO_2_, heavy metals, polar dyes, drugs, toxins, or endocrine-disrupting chemicals (EDC).^20–25^ HCLRs can be obtained in the form of thin polymeric membranes^26^ or nanocomposites containing different functional nanofillers, such as magnetic nanoparticles,^27^ carbon nanotubes,^28^ or graphene oxide.^29^ Moreover, these microporous materials can be combined with different macroporous materials in order to synergistically merge their adsorption properties, creating hierarchically porous materials.^30^ The hyper-cross-linking approach has also been pursued to generate novel porosity into the porous channels and onto the external surface of mesoporous silica nanoparticles (MSN), with the aim to enhance their adsorption properties.^31^

Despite the excellent properties mentioned above, the monomers that are usually exploited in the synthesis of these HCLRs are mainly fossil-based. In the past few years, several biobased adsorbents have been realized through different physical/chemical modifications of biochar, agricultural wastes, rocks and minerals, rice husks, and algae.^32–34^ Furthermore, cellulose is a very interesting biomaterial. Cellulose is a natural polymer considered as a valuable alternative to synthetic polymers in various applications due to its excellent thermal stability, chemical resistance, mechanical strength, nontoxicity, and biodegradability, in addition to being an indisputably large renewable resource and the most abundant organic compound on earth.^35^ Cellulose nanofibrils (CNF), long, semicrystalline, and flexible fibrils, are interesting materials for adsorption due to their small diameters that make their SSA considerably high.^36^ Thanks to the abundance of functional groups available on its surface and in order to enhance its adsorption capacity, cellulose is often chemically modified through various functionalization approaches. For example, succinylated, organosilylated, thiolated, phosphorylated, acetylated, or amino-functionalized nanocelluloses were realized.^37^ An additional method worth mentioning for the purpose of this work is the grafting of polymers on the surface of cellulose. Polymer grafting is mainly achieved by exploiting the reactivity of the hydroxyl groups on the cellulose surface.^37–39^ In addition, as reported by Kaya,^40^ cellulose can be cross-linked with nontoxic weak acids (such as citric acid) to form a macroporous three-dimensional network.

In this work, a novel strategy to realize microporous adsorbents for water remediation applications was designed and validated. This study proposes the hypothesis that the functionalization of cellulose nanofibrils with an aromatic organic phase exploitable for hyper-cross-linking is a valid strategy to generate a micro/mesoporous shell on CNF and obtain a high surface area material mostly composed of cellulose nanofibrils with reduced fossil-based content. The functionalized CNF have a significantly enhanced adsorption capacity toward aromatic pollutants through physisorption. This approach will yield a versatile adsorbent for aromatic pollutants with no specificity over their molecular charge, and this route has never been pursued in the literature, which is mostly focused on cellulose functionalization to promote specific interactions with target pollutants such as metal ions or cationic dyes.^35–39^ After in-depth characterization of the functionalized nanofibrils’ chemophysical properties, the adsorption capacity of the as-developed nanomaterial toward a halogenated aromatic pollutant (2,4-dichlorophenol (DCP)) was tested through equilibrium and kinetic adsorption tests. The thermodynamic parameters of enthalpy, entropy, and Gibbs free energy change were evaluated through an adsorption test at variable temperatures.

## 2 Experimental Section

### 2.1 Materials

Cellulose nanofibrils (CNF, catalogue code Celova M250R-P, 10 wt % in water) were kindly provided by Weidmann Fiber Technology-Weidmann Electrical Technology AG (Rapperswil, Switzerland). Citric acid (CA, ≥99.5%), sodium hydroxide (NaOH, ≥99.0%), vinylbenzyl chloride (VBC, ≥95.0%, mixture of isomers, ∼70% meta + ∼30% para), toluene (99.8%), 2,2′-azobis­(2-methylpropionitrile) (AIBN, >98%), tetraoctylammonium bromide (TOAB, >98%), 1,2-dichloroethane (DCE, ≥99.0%), tetrahydrofuran (THF, ≥99.0%), chloroform (≥99.8%), FeCl_3_ (≥97%), 2,4-dichlorophenol (DCP, 99%), and humic acid (HA) were purchased from Sigma-Aldrich (Milan, Italy) and used without further purification.

### 2.2 Cellulose Nanofibril Functionalization

Cellulose nanofibrils were first carboxylated via esterification with citric acid. CNF were treated with an 80 wt % water solution of citric acid. First, CA was dissolved in a flask under reflux. Then, the CNF water dispersion was added to the flask, and the suspension (1 wt % CNF) was kept under stirring at 100 °C to promote the carboxylation reaction. Samples were collected after 2, 4, 24, and 48 h. The carboxylated CNF were neutralized through subsequent washing with distilled water and centrifugation (8000 rpm, 15 min, 4 °C) using an Avanti J-25 centrifuge (Beckman Coulter). The resulting samples were coded CNF_*n*h, where *n* indicates the reaction time (e.g., CNF_2h)

Then, poly­(vinylbenzyl chloride) was synthesized ex situ in order to graft it on CNF. In detail, VBC (10 mL) was poured into a 250 mL round-bottom flask containing 20 mL of toluene and kept under stirring for 30 min in a N_2_ atmosphere. 100 mg of AIBN was added, the solution was heated to 60 °C, and the polymerization reaction was carried out under reflux for 14 h in a N_2_ atmosphere. The as-obtained poly­(vinylbenzyl chloride) (PVBC) was precipitated in ethanol, filtered, and dried at 40 °C under vacuum overnight.

Among carboxylate CNF samples, CNF_24h was surface-functionalized by grafting with PVBC by the phase transfer reaction. 600 mg of CNF_24h was added to 350 mL of NaOH aqueous solution (0.005 M) in a 1 L round-bottom flask and stirred for 15 min at room temperature. Then, 1 g of TOAB was added to the flask, and the solution was heated in an oil bath at 60 °C. Separately, 300 mg of PVBC was first dissolved in 250 mL of toluene under stirring and then added to the main flask with the cellulose dispersion. The reaction was carried out under stirring for 22 h. Then, the mixture was transferred into a separatory funnel, in which it was separated into two phases, one water-based and the other toluene-based, with cellulose localizing at the interface. The functionalized cellulosic product (coded CNF-PVBC) was separated, washed with chloroform and THF, and separated by centrifugation (13 500 rpm, 4 °C, 10 min) with a Z326 K centrifuge (Hermle Labortechnik). Finally, THF was exchanged with 1,2-dichloroethane (DCE) for the hyper-cross-linking reaction.

Finally, the hyper-cross-linking of the PVBC phase in CNF-PVBC was promoted by the Friedel–Crafts reaction. 400 mg of CNF-PVBC was mixed with 40 mL of DCE under nitrogen for 2 h in a round-bottom flask at room temperature. Then, the mixture was cooled to 4 °C in an ice–water bath, and 2.1 g of FeCl_3_ was added to the mixture, which was mixed for 2 h. Then, the flask was heated to 80 °C in an oil bath, and the reaction was carried out for 18 h, under reflux, in a N_2_ atmosphere with continuous stirring. The hyper-cross-linked material (coded xCNF-PVBC) was washed with methanol, separated by centrifugation (13 500 rpm, 10 °C, 10 min) using a Z326 K centrifuge, and dried under vacuum at 60 °C.

### 2.3 Characterization

Fourier transform infrared (FTIR) spectroscopy was performed on CNF, CNF_2h, CNF_4h, CNF_24h, CNF_48h, CA, CNF-PVBC, and xCNF-PVBC before and after adsorption of DCP using a PerkinElmer Spectrum One spectrometer equipped with an attenuated total reflectance (ATR) module, using a resolution of 4 cm^–1^ and 32 scan collections in the ATR mode. All samples were dried in an oven under vacuum at 60 °C prior to analysis. The spectra were recorded in the 4000–650 cm^–1^ range.

CNF_2h, CNF_4h, and CNF_24h were quantitatively analyzed by titration to determine the COOH concentration on the surface of the treated CNF. In a typical experiment, the pH of the carboxylated CNF dispersions (2 mg/mL in distilled water) was adjusted to 2.5 using a 0.1 M HCl solution. Then, the mixtures were titrated with NaOH solution (0.1 M) up to pH 9 in order to surpass the equivalence point and calculate the concentration of the carboxylates on CNF. Measurements were performed in triplicate, and the average values and standard deviation were evaluated.

Thermogravimetric analysis (TGA) was carried out on CA, CNF, CNF_2h, CNF_4h, CNF_24h, CNF-PVBC, and xCNF-PVBC with a PerkinElmer Pyris 1 TG/DTA analyzer, using nitrogen as purge gas (40 mL/min). About 3 mg of sample was heated from 25 to 800 °C at 10 °C/min. In order to eliminate all the water content, the samples were held at 100 °C for 10 min before the heating continued.

Morphological analysis of CNF, CNF_2h, CNF_4h, CNF_24h, CNF-PVBC, and xCNF-PVBC was performed by transmission electron microscopy (TEM) and scanning electron microscopy (SEM).

For transmission electron spectroscopy (TEM), the copper grips were immersed in diluted dispersions of the samples. The analysis was performed using an FEI Tecnai G12 Spirit Twin (LaB6 source) at a 120 kV acceleration voltage (FEI, Eindhoven, The Netherlands). TEM images were collected with an FEI Eagle 4k CCD camera.

Scanning electron microscopy (SEM) was carried out using an FEI Quanta 200 FEG SEM. Prior to the analysis, the samples were freeze-dried to avoid agglomeration of the cellulose fibers and deposited on aluminum stubs by means of carbon adhesive disks. Samples were sputter-coated with a thin layer of Au/Pd and observed with a secondary electron detector at an acceleration voltage of 10–30 kV.

CNF, CNF_24h, CNF-PVBC, and PVBC were analyzed by solid-state ^13^C cross-polarization (CP) magic angle spinning (MAS) NMR using a Bruker Avance II 400 spectrometer operating at a static field of 9.4 T, equipped with a 4 mm MAS probe, using a ^1^H π/2 pulse width of 3.6 μs, a contact time of 2 ms, and a repetition time of 5 s. Finely ground samples were packed in 4 mm zirconia rotors sealed with Kel-F caps and spun between 11 and 13 kHz.

The ζ potential of CNF and CNF_24h was determined on 2 mg/mL dispersions by laser Doppler microelectrophoresis, using a Zetasizer Nano ZS (Malvern Instruments, U.K.). Measurements were performed at 25 ± 0.1 °C. The ζ potential was calculated as the average of 3 measurements, where each measurement comprised 20 subruns.

Gas adsorption volumetric analysis was performed on xCNF-PVBC and CNF by using a Micromeritics 3Flex analyzer. Nitrogen adsorption/desorption isotherms were collected at 77 K, and the SSA was determined from the linear part of the Brunauer–Emmett–Teller (BET) equation. The total pore volume was determined from the N_2_ adsorption isotherms at a *p*/*p* ^0^ of 0.97. Nonlocal density functional theory (NLDFT) was applied to the N_2_ adsorption isotherms to evaluate the pore size distribution of the materials. Prior to the analysis, all samples were degassed at 120 °C under vacuum (*P* < 10^–7^ mbar). All adsorption measurements were performed using high-purity gases (>99.999%).

### 2.4 Adsorption of DCP from Solution

xCNF-PVBC and CNF were tested for the adsorption of DCP from water. Prior to analyses, the samples were dried in an oven under vacuum. Kinetic and equilibrium adsorption tests were performed. A constant 1 mg/1 mL sorbent/solution ratio was kept for all samples. For xCNF-PVBC, about 7 mg of sorbent and 7 mL of DCP solution were employed for the tests; for CNF, double doses were employed in order to minimize possible weight errors of the highly hydrophilic CNF. All measurements were performed in triplicate. For the kinetic studies, CNF and xCNF-PVBC were introduced into vials containing DCP at 62.5 mg/L and kept at 298 K. The supernatant solution was collected at regular time intervals and analyzed by ultraviolet (UV) spectroscopy using a Jasco V570 UV spectrometer. Through a prerecorded calibration curve of DCP in water, the DCP concentration in the supernatant solution was determined. Pseudo-first-order (PFO) and pseudo-second-order (PSO) models were applied. Equilibrium adsorption tests were performed at 298, 308, 318, and 328 K at various concentrations of DCP ranging from 15 to 1000 mg/L. CNF and xCNF-PVBC samples were introduced into vials containing the DCP solutions, and the vials were kept at constant temperature until equilibrium was reached. Therefore, the DCP concentration in the supernatant was evaluated through UV spectroscopy. The Freundlich and Langmuir models were employed to fit the results. The enthalpy, entropy, and Gibbs free energy changes were evaluated.

Recycling tests were conducted on the xCNF-PVBC to test the regeneration efficiency. Similar to the previous tests, about 7 mg of sorbent and 7 mL of DCP solution were employed. A DCP solution at 62.5 mg/L was used, and the adsorption was conducted at 298 K. Five adsorption cycles were performed on the same sample. Adsorption was promoted by mixing to accelerate the achievement of equilibrium, and UV analysis of the supernatant solution was performed after 24 h. After adsorption, the sample was regenerated by five washes at 60 °C with approximately 15 mL of ethanol. Once the adsorbent was purified, it was dried under vacuum and reanalyzed for subsequent adsorption. The adsorption efficiency was evaluated as the percent ratio of the adsorption capacity of the *n*th cycle and the first cycle. The tests were conducted in triplicate.

The effect of pH on DCP adsorption was investigated by evaluating the equilibrium adsorption capacity of xCNF-PVBC in a DCP solution at 62.5 mg/L at variable pH values (3, 5, 7, 9, 11). The effect of the presence of HA in water on the adsorption of DCP was evaluated by testing the adsorption capacity of DCP by xCNF-PVBC in a water solution containing both DCP and HA. Six tests were conducted with different DCP and HA concentrations. The DCP concentrations were fixed at 62.5 and 1000 mg/L, and those of HA were fixed at 2, 10, and 50 mg/L. Adsorption was promoted by mixing, and UV analysis of the supernatant solution was performed after 24 h. The efficiency was evaluated as the percent ratio of the DCP adsorption capacity in the presence of HA and in the absence of HA. All tests were conducted in triplicate.

## 3 Results and Discussion

In this work, cellulose was proposed as an environmentally friendly alternative to the state-of-the-art materials commonly used for the treatment of polluted water through physisorption. In order to obtain a functional material for the proposed application, the following approach was followed. First, the cellulose nanofibrils were carboxylated using a weak acid (citric acid) to allow further functionalization with poly­(vinylbenzyl chloride) (Figure 1a). Then, poly­(vinylbenzyl chloride) was grafted onto the surface of the CNF through nucleophilic substitution of the alkyl halide (Figure 1b). Finally, the PVBC-grafted CNF were hyper-cross-linked through Friedel–Crafts alkylation (Figure 1c). The high degree of cross-linking leads to the formation of micro/mesoporosity and so to the formation of a high surface area material.

**Figure 1:**
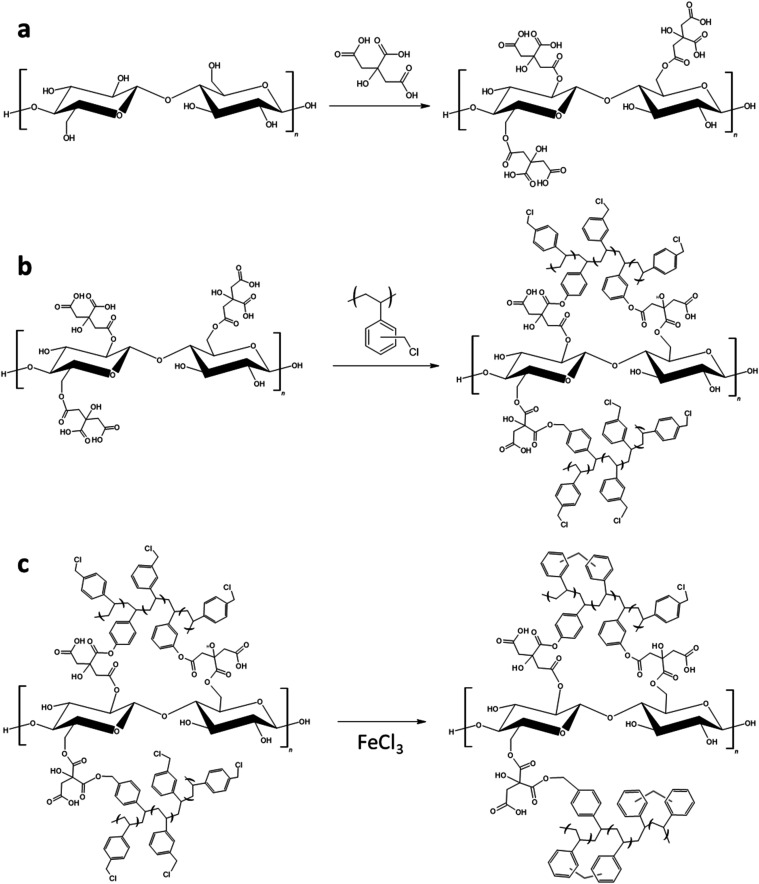
Scheme of the carboxylation process (a), PVBC grafting (b), and hyper-cross-linking reaction (c).

### 3.1 CNF Carboxylation

The degree of carboxylation of the cellulose nanofibrils was investigated through FTIR spectroscopy, titration analysis, and TGA.

By ATR-FTIR analysis of CNF and the carboxylated cellulose nanofibrils, the kinetics of citric acid grafting onto cellulose were determined (Figure 2a,b). The CNF spectrum shows the typical polysaccharide stretching vibrations of O–H and C–H bonds between 3660 and 2900 cm^–1^; the wide peak at 3331 cm^–1^ indicates the hydroxyl group stretching vibration in polysaccharides, which includes hydrogen bond vibrations both inside and between molecules in cellulose; the C–H stretching vibrations signal is centered at 2894 cm^–1^; the region between 1630 and 900 cm^–1^ is rich of typical bands attributed to cellulose; the vibration of water molecules absorbed is shown by the peak at 1633 cm^–1^; the absorption bands located at 1428, 1367, 1334, 1027, and 896 cm^–1^ are associated with the stretching and bending vibrations of cellulose −CH_2_, −CH, −OH, and C–O bonds; the band located at approximately 1420–1430 cm^–1^ is indicative of the fraction of crystalline cellulose, whereas the band located at 897 cm^–1^ is indicative of the amorphous portion of cellulose.^41^

**Figure 2:**
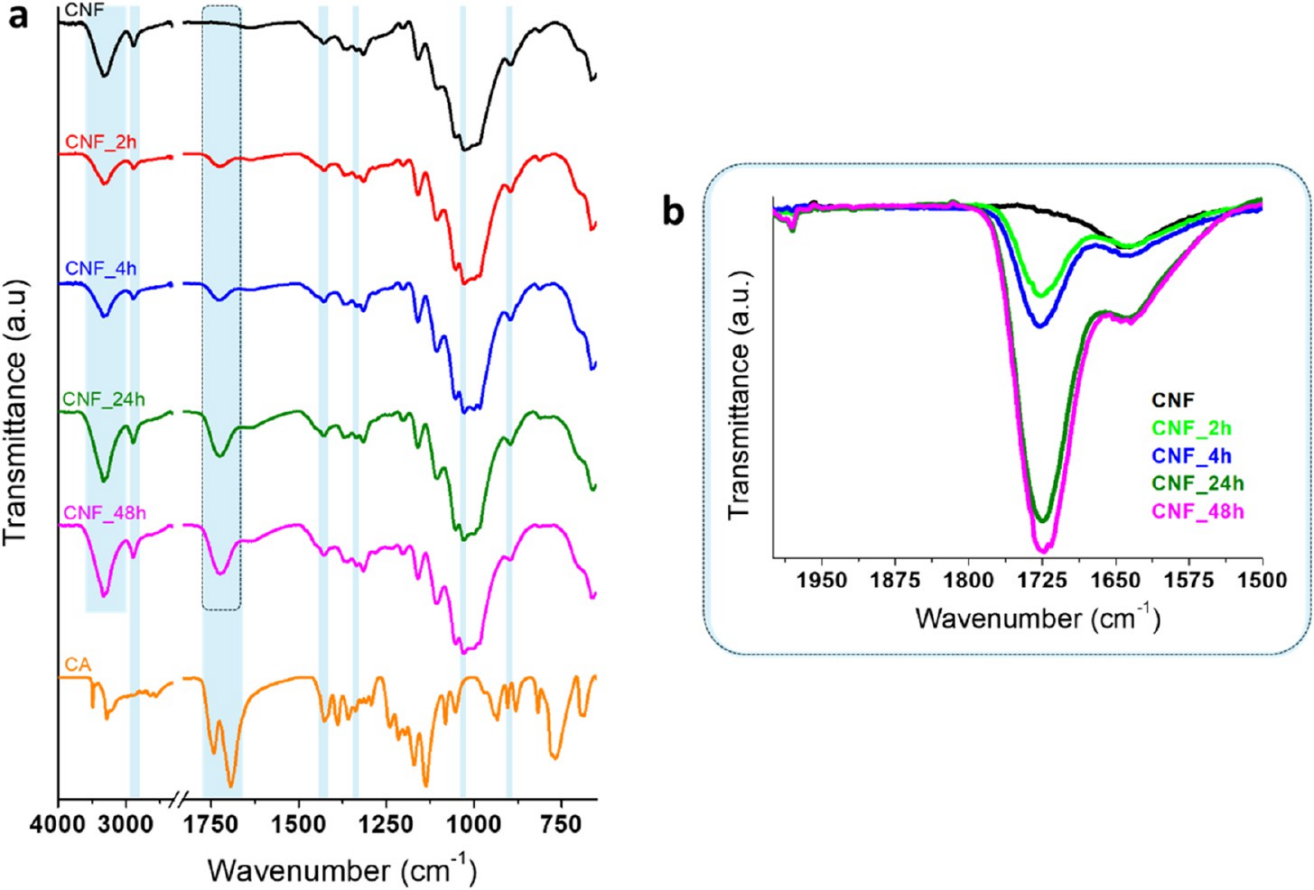
FTIR spectra of CNF, CNF_2h, CNF_4h, CNF_24h, CNF_48h, and CA (a), magnification of (a) showing the 2000–1500 cm^–1^ region of CNF and carboxylated CNF spectra (b).

After carboxylation, the oxidized samples show the typical absorption signals of cellulose and, in addition, the characteristic absorption band of the carbonyl group due to the stretching of the C=O bond centered at 1725 cm^–1^ ^42,43^ (Figure 2a,b). No signal appeared at the same position for pristine CNF. The results show that the amount of carbonyl groups in the carboxylated CNF increases with the reaction time (Figure 2b). Indeed, the esterification of citric acid with cellulose, other than being dependent on the acid concentration, is significantly dictated by the reaction time.^44^ In particular, the sample CNF_24h exhibits a significantly more pronounced carbonyl signal with respect to CNF_4h, while prolonging the reaction up to 48 h (CNF_48h) does not induce a further significant intensification of the band centered at 1725 cm^–1^. Therefore, considering the much longer reaction time of CNF_48h and the fact that CNF_48h and CNF_24h spectra are almost comparable, the 48 h reaction time was discarded, and sample CNF_48h was not considered for further analyses.

Titration analyses performed on CNF_2h, CNF_4h, and CNF_24h yielded results that were highly consistent with the FTIR analysis. Specifically, the content of carboxylic groups detected in the first two samples is very similar, approximately 13.9 ± 0.4 wt %, while CNF_24h shows an increase in −COOH group content, reaching a value of 16.5 ± 0.5 wt %. Thus, FTIR and titration analyses indicate that there are no significant differences between the reaction times of 2 and 4 h, whereas a substantial increase in the number of carboxylic groups is observed at 24 h. This was evidenced both by a significant enhancement of the C=O FTIR peak and by an increase of approximately 23% in the −COOH group content determined by titration. Extending the reaction time by an additional 24 h does not lead to a further significant increase in the carbonyl peak intensity; therefore, this sample was not subjected to titration analysis, also considering the considerably longer duration of this 48 h reaction and thus its associated lower energy sustainability.

The thermal properties of CNF, CNF_2h, CNF_4h, and CNF_24h were evaluated through TGA. Figure 3a also shows the TGA trace of citric acid, as a comparison, conducted in a nitrogen atmosphere. As shown in the figure, the degradation temperature of citric acid is significantly lower than that of CNF. Accordingly, the carboxylated samples show the anticipated degradation onset with respect to CNF, as shown by the characteristic temperatures of 5 and 50 wt % weight loss (T_5_ and *T*_50_, respectively, Figure 3b). Indeed, *T*_5_ decreases by about 26 °C for CNF_2h and CNF_4h with respect to CNF, and by about 43 °C for CNF_24h, due to the more consistent carboxylation extent. The 50% weight loss temperature values are very close for all the cellulosic samples, being determined for the most by the cellulose contribution. As for the residual weight at 800 °C, it is important to note that the trend could be against intuitive since citric acid has a lower residual weight than cellulose at 800 °C. However, samples of CNF reacted with citric acid show higher char residues than CNF, which might be due to partial cross-linking of CNF due to the formation of strong covalent ester bonds with CA. Indeed, CA is a multifunctional acid containing three −COOH groups, whose condensation with multiple hydroxyl groups of adjacent cellulose fibrils can lead to the formation of a cross-linked network. However, in the diluted water suspension conditions used to carry out the carboxylation reaction, this is likely to take place just in a moderate extent.^40,44^

**Figure 3:**
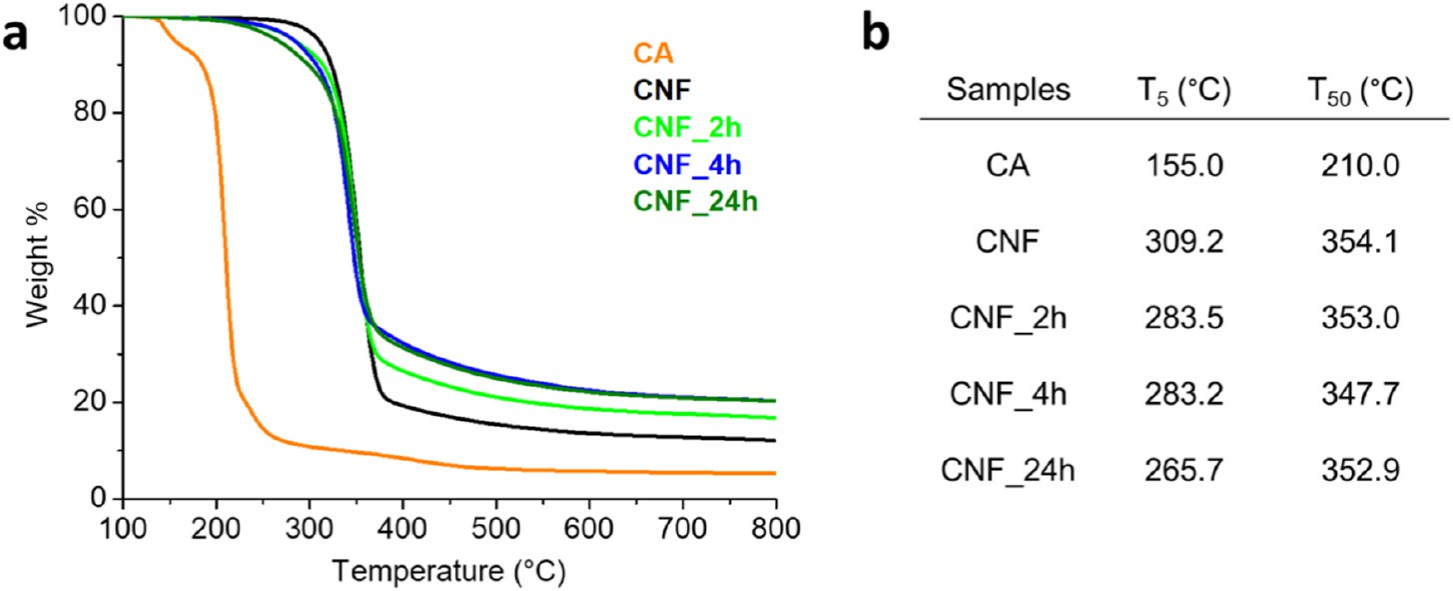
TGA traces in a nitrogen atmosphere (a), and 5 and 50 wt % degradation temperatures (b) of CA, CNF, CNF_2h, CNF_4h, and CNF_24h.

The morphologies of CNF and carboxylated samples CNF_2h, CNF_4h, and CNF_24h were analyzed by transmission electron microscopy (Figure 4). The TEM images show that the esterification reaction did not significantly affect the fiber morphology. CNF and the carboxylated samples are all constituted of long fibrils (tens of micrometers in length) with a diameter range of 10–200 nm.

**Figure 4:**
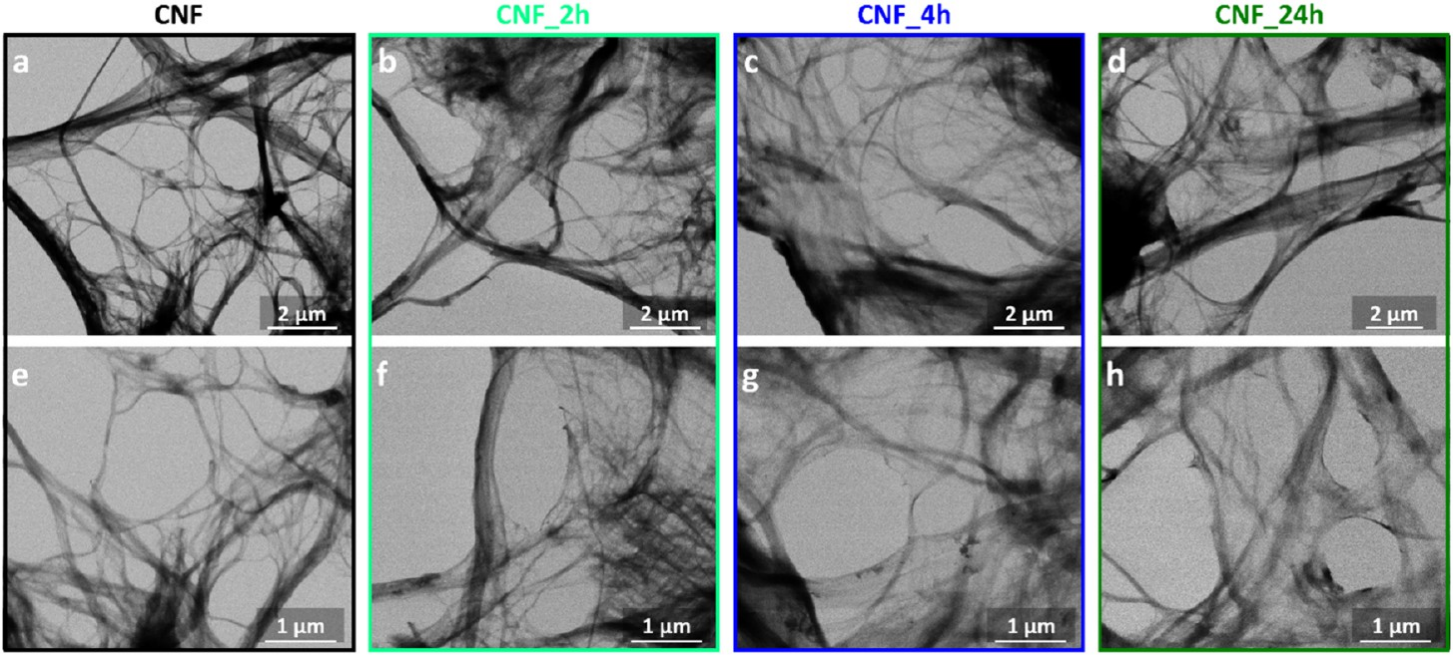
TEM images of CNF (a, e), CNF_2h (b, f), CNF_4h (c, g), and CNF_24h (d, h).

CNF, CNF_2h, CNF_4h, and CNF_24h were also observed by SEM (Figure 5). Dry samples were obtained by freeze-drying the cellulose nanofibril aqueous dispersions at 0.7 wt % concentration. All samples show a three-dimensional interconnection of the cellulose nanofibrils. In particular, CNF, CNF_2h, and CNF_4h exhibit diffuse lamellar structures in their network, while the most functionalized sample, CNF_24h, exhibits a more homogeneously interconnected network of cellulose nanofibrils.

**Figure 5:**
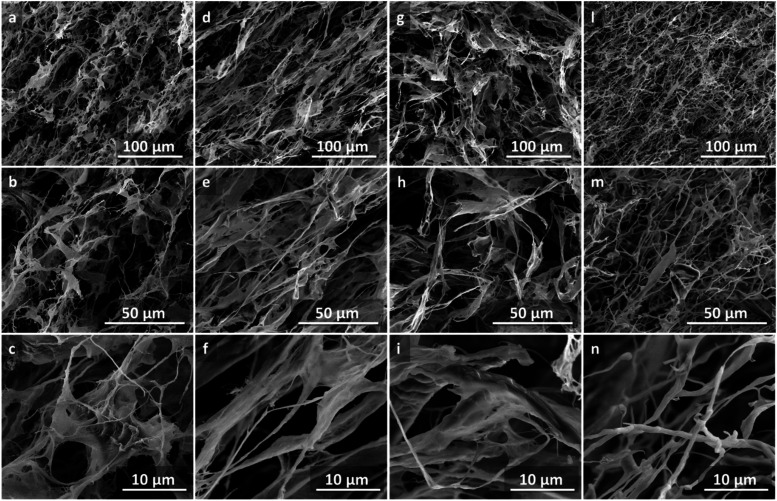
SEM images of CNF (a–c), CNF_2h (d–f), CNF_4h (g–i), and CNF_24h (l–n).

Considering all the results, the 24 h reaction time sample was selected as a precursor for the grafting process of PVBC, in which the carboxylic groups are exploited to graft the polymer phase to the cellulosic chain. Indeed, CNF_24h showed a significantly higher carboxylation extent, and the thermal and surface properties of this sample were not modified to a great extent. Sample CNF_24h was then analyzed by NMR and ζ potential analysis.

Through ^13^C solid-state NMR, the grafting yield of citric acid was evaluated. Such evaluation was performed by calculating the ratio between the area of the peak centered at 172 ppm, attributed to carbon atoms of the carboxyl group of CA, and the area of the peak centered at 105 ppm, assigned to C1 carbon atoms of the glucose units of cellulose (Figure S1). The results indicate 9.45 C=O groups per 100 C1 carbon atoms. Considering that each CA unit included 3 carboxyl groups, a grafting of 3.15 mol of CA per 100 mol of glucose units is obtained.

ζ potential analysis of CNF and CNF_24h suspensions showed values of −18.7 ± 0.2 and −31.6 ± 0.3 mV, respectively. The significant increase of the absolute value of the electrical double layer of the charged particles in water is due to the presence of added carboxyl functional groups and is indicative of the higher colloidal stability of CNF_24h with respect to CNF.^45^

### 3.2 Grafting of CNF with PVBC

The grafting reaction of PVBC onto carboxylated cellulose is schematized in Figure 1b. CNF_24h was previously dispersed in NaOH solution. The reaction between the obtained carboxylate salts, CNF-COO^–^, in the aqueous phase, and PVBC, dissolved in toluene, occurred through nucleophilic substitution of CNF-COO^–^ to replace the chloride leaving group of PVBC and was promoted by the phase transfer catalyst TOAB.^28^

This process distinctly changes the morphology of the cellulose nanofibrils. The SEM images of CNF-PVBC (Figure 6a–c) show the presence of a prominent polymeric phase covering the cellulose nanofibrils, although the cellulose nanofibril features are clearly visible, protruding from the grafted polymer phase. CNF-PVBC observed by transmission electron microscopy also shows the presence of a lower contrast coating on cellulose fibrils, which can be attributed to the grafted polymer phase (Figure 6c,d).

**Figure 6:**
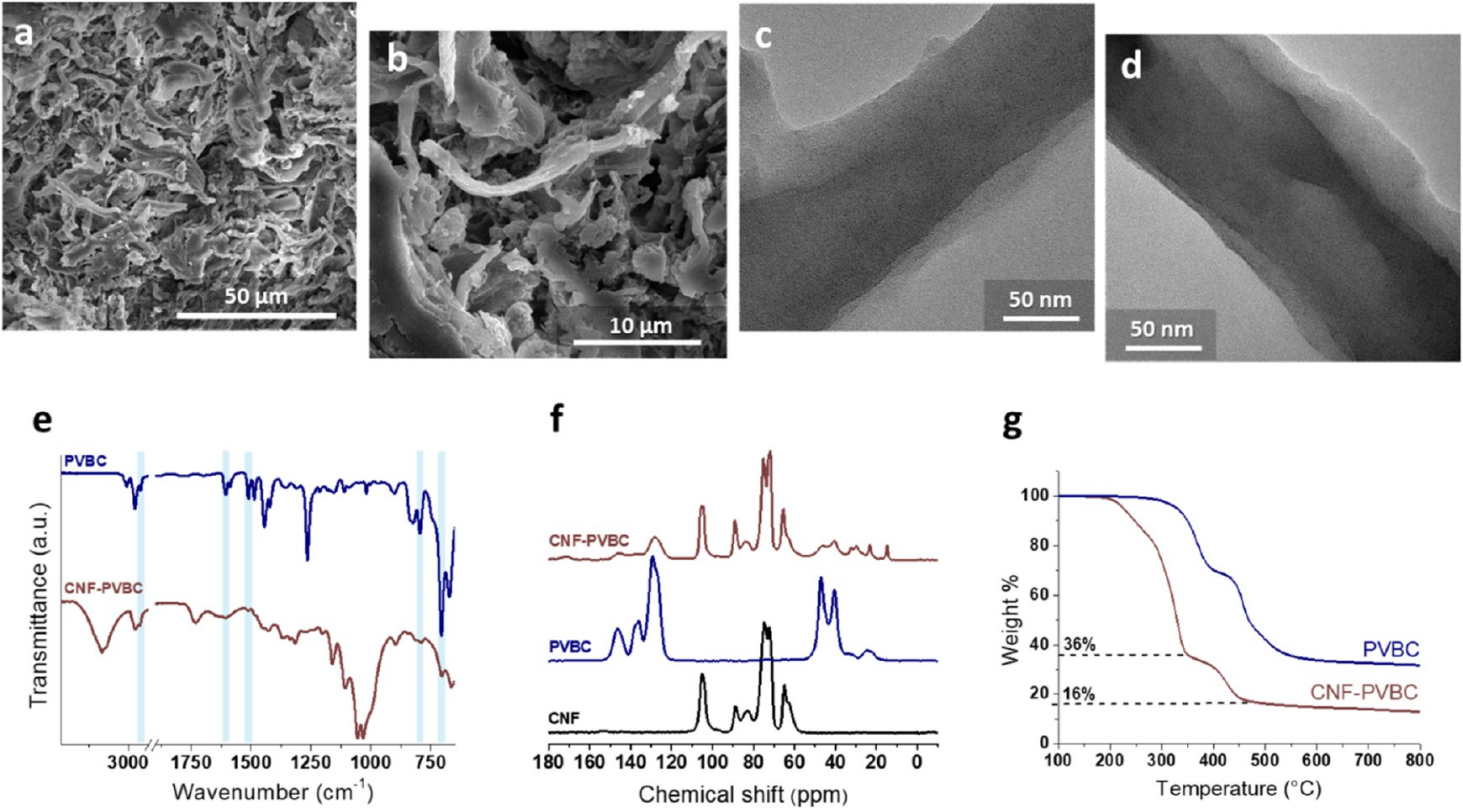
CNF-PVBC SEM (a, b) and TEM (c, d) images, ATR-FTIR spectra of PVBC and CNF-PVBC (e), ^13^C CP MAS NMR spectra of CNF-PVBC, PVBC, and CNF (f), and TGA traces of PVBC and CNF-PVBC in a nitrogen atmosphere (g).

The product of the grafting reaction was also characterized by FTIR, NMR, and TGA analysis. Figure 6e shows the FTIR spectra of PVBC and CNF-PVBC. The CNF-PVBC spectrum shows both typical signals of cellulose (CNF_24h) and the characteristic peaks of PVBC. The latter, at wavenumbera 2920, 1608, 1509, 794 and 705 cm^–1^, are marked in Figure 6e with vertical bars.

^13^C CP MAS NMR analysis was conducted on CNF-PVBC, PVBC, and CNF in order to establish the amount of polymer grafted onto CNF (Figure 6f). The main PVBC and CNF signals fall within different frequency ranges, allowing the identification of the NMR signals attributed to each contribution in the sample CNF-PVBC. In PVBC, peaks at lower fields (160–120 ppm) refer to the aromatic carbons, while peaks at higher fields (55–35 ppm) are related to the aliphatic backbone and to the −CH_2_–Cl methylene. CNF and PVBC signals are both present in the CNF-PVBC spectrum, highlighting the occurrence of the grafting reaction. In particular, since these spectral regions do not overlap, by comparing the regions attributable to the PVBC phase and the CNF phase with their respective reference spectra, it is possible to estimate the relative content of the PVBC and CNF phases, which is 30% PVBC and 70% CNF by weight. It is interesting to note that the signal at 47 ppm, related to the −CH_2_–Cl group, decreases in intensity in CNF-PVBC with respect to the PVBC spectrum. This can be ascribed to the partial reaction of these groups during the grafting of PVBC onto the CNF.

The TGA results are shown in Figure 6g. The PVBC thermogram exhibits two major degradation steps, the first from about 220 °C up to 414 °C and the second from about 415 to 630 °C. The CNF-PVBC thermogram shows a major degradation step from about 130 °C up to 369 °C and a second step from about 370 to 510 °C. From the analysis of the degradation temperature of carboxylated cellulose CNF_24h (Figure 3a) and PVBC, it may be concluded that, while in the first degradation step of CNF-PVBC, the degradation of both the cellulosic fibrils and the polymer moieties may be involved, in the second step, only the PVBC moieties are involved. Therefore, accurate analysis of the grafted PVBC phase is not possible based solely on TGA analyses. However, the weight loss in the 370–510 °C step is about 20 wt %; considering that part of the polymer phase is also degraded in the first step (130–369 °C), the TGA results confirm that the grafted amount of PVBC exceeds 20 wt %, in compliance with the NMR results.

### 3.3 CNF-PVBC Hyper-Cross-linking

The hyper-cross-linking reaction of CNF-PVBC is schematized in Figure 1c. CNF-PVBC is dispersed in a swelling solvent (DCE) for the polymer phase; then, Friedel–Crafts alkylation is promoted, and the internal cross-linker (PVBC) reacts, forming methylene bridges between neighboring aromatic rings of the resin (schematized in red in Figure 1c). xCNF-PVBC has the appearance of a brownish powder, as shown in Figure 7a.

**Figure 7:**
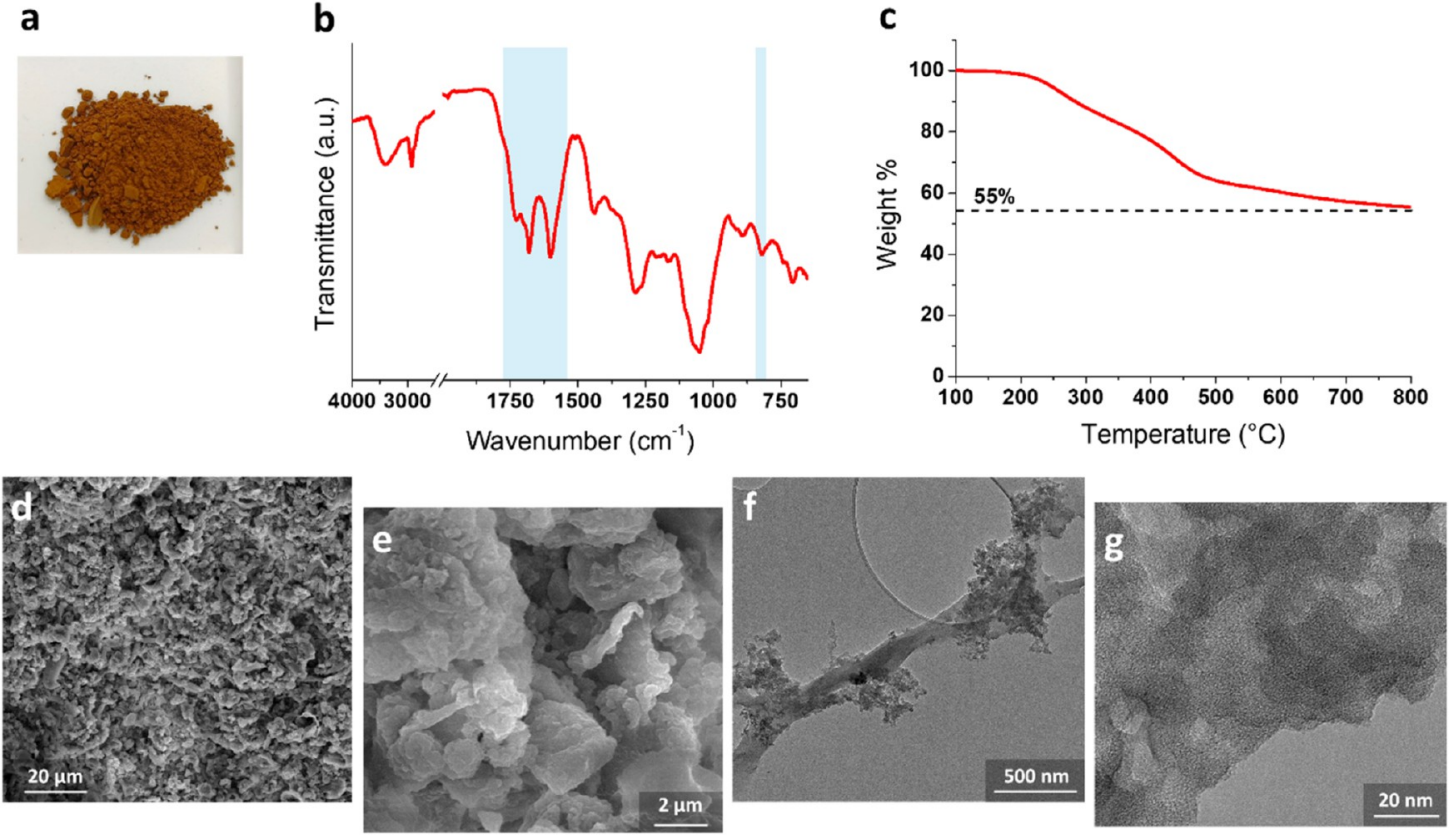
Photographic image (a), ATR-FTIR spectrum (b), TGA trace in a nitrogen atmosphere (c), SEM (d, e), and TEM (f, g) images of xCNF-PVBC.

Hyper-cross-linking was confirmed by FTIR analysis (Figure 7b). A significant difference between the FTIR spectra of the hyper-cross-linked material xCNF-PVBC and the corresponding precursor (Figure 6e) is the intensification of the xCNF-PVBC absorption band centered at about 805 cm^–1^. This band is attributed to disubstituted aromatic groups, confirming the occurrence of the hyper-cross-linking reaction. Moreover, in the xCNF-PVBC spectra, a complex absorption band in the wavenumber range 1650–1780 cm^–1^ is also observed; this band, often recorded in hyper-cross-linked resins,^28^ has been well discussed by Tsyurupa et al.,^46^ who attributed it to hindered vibrations of carbon–carbon bonds and valence angles in aromatic rings. Finally, the general broadening of the peaks present in the xCNF-PVBC spectrum is further attributed to the hyper-cross-linked nature of the sample.

The TGA results, shown in Figure 7c, highlight the increased thermal stability of xCNF-PVBC with respect to that of CNF-PVBC (Figure 6g). xCNF-PBVC is characterized by a residue at 800 °C above 50 wt %, which is significantly higher than that of CNF-PVBC (about 14%). This is attributed to the highly cross-linked structure of the functionalized cellulose nanofibrils, which confers them higher thermal resistance.

SEM images of xCNF-PVBC are shown in Figure 7d,e. Compared to CNF-PVBC (Figure 6a,b), xCNF-PVBC exhibits a more open structure, which is especially noticeable at low magnification (Figure 7d). Beyond that, it is noteworthy that the cellulose structure is still preserved, as indicated by the characteristic morphology of the cellulose fibers visible in Figure 7e (SEM),f (TEM). The TEM images also show that xCNF-PVBC exhibits widespread porosity in the grafted polymer phase. However, for amorphous micro/mesoporous materials, such as the one presented in this study, it is not possible to estimate the pore size distribution based on the TEM morphology. The pore size distribution of xCNF-PVBC was analyzed through N_2_ adsorption analysis, and CNF were also analyzed for comparison. The adsorption–desorption isotherms for N_2_ at 77 K and the NLDFT pore size distribution graphs are shown in Figure 8. The BET-SSA, total pore volume, and porosity data are presented in Table 1.

**Figure 8:**
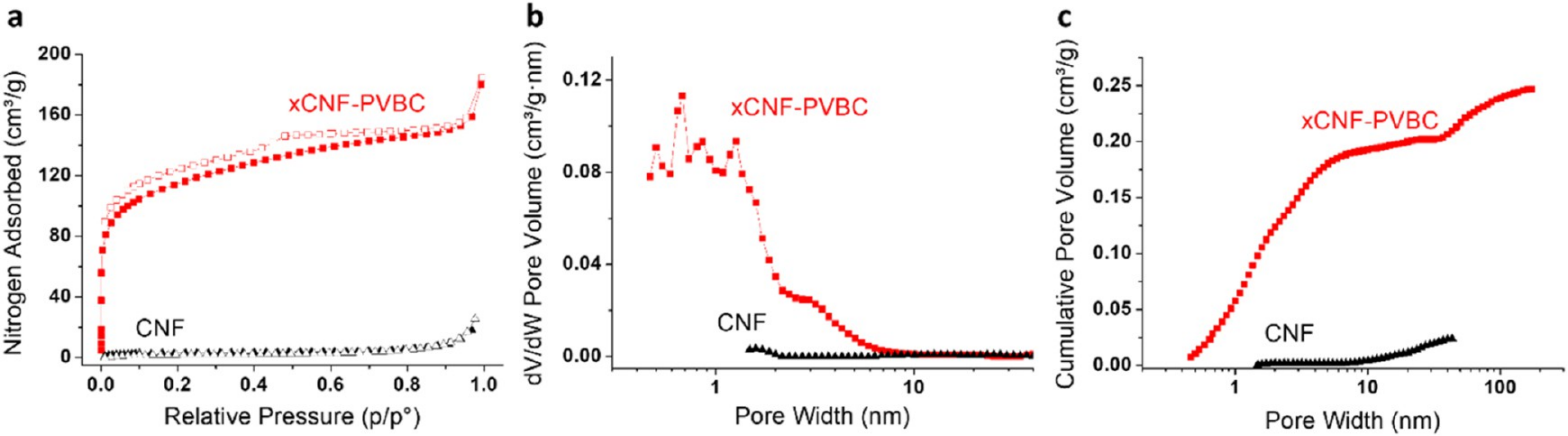
CNF and xCNF-PVBC nitrogen adsorption/desorption isotherms at 77 K (adsorption in full symbols, desorption in empty symbols) (a) and NLDFT pore size distribution (b, c).

**Table 1:** Textural Properties of xCNF-PVBC and CNF

sample	BET-SSA (m^2^/g)	total pore volume (cm^3^/g)	microporous fraction (%)
xCNF-PVBC	408.7 ± 2.8	0.246	50.1
CNF	10.9 ± 0.1	0.028	

The xCNF-PVBC isotherm is a combination of type I and type II isotherms with a moderate hysteresis in desorption; xCNF-PVBC shows significant N_2_ adsorption at a very low relative pressure, attesting to the presence of a significant microporous fraction. Indeed, the NLDFT pore size distribution of xCNF-PVBC shows that the sample is characterized prevalently by micro- and mesoporosity (Figure 8b,8c), with a microporous fraction of about 50% and SSA of about 409 m^2^/g (Table 1). CNF nitrogen adsorption was also analyzed for comparison. Pristine CNF is characterized by an isotherm resembling a type III isotherm, typical of macroporous or nonporous materials. Indeed, the BET SSA of CNF is about 10 m^2^/g, and the NLDFT pore size distribution shows the nonporous nature of the material. The very low value of porosity attributed to CNF is ascribed to the interfibrillar porosity.

### 3.4 Adsorption from an Aqueous Solution

Adsorption tests were performed using 2,4-dichlorophenol as an organic pollutant. First, solutions were prepared at known concentrations of the above pollutant from 15 mg/L up to 1000 mg/L. The solutions were analyzed by UV spectroscopy, and the absorbance of the peak centered at 284 nm was measured and used to build the calibration curve for DCP. The concentration of the test solutions was obtained through the Lambert–Beer law:

1 A=εlC

where *A* is the absorbance, ε (M^–1^ cm^–1^) is the molar extinction coefficient derived from the calibration curve, *l* (cm) is the optical path length, and *C* (mg/L) is the concentration of the solution. Kinetic tests of adsorption were performed at *C*_0_ 62.5 mg/L and at 298 K for xCNF-PVBC (Figure 9b) and CNF (Figure S2a). The fit with the pseudo-first-order and pseudo-second-order models was also evaluated, and the parameters are reported in Table S1. In both equations, *q*_*t*_ is the adsorbed amount of DCP at time *t*, *q*_e_ is the adsorption capacity at equilibrium, and *k*_1_ and *k*_2_ are the first- and second-rate constants, respectively. The pseudo-first-order (PFO) kinetics is regulated by the following equation

2 $$q_{t}=q_{e}\left(1-e^{-k_{1}t}\right)$$

and assumes that the control of the adsorption rate depends on the diffusion of the adsorbate on the adsorbent surface. The pseudo-second-order (PSO), following the equation

3 $$q_{t}=\frac{q_{e}^{2}q_{t}t}{1+k_{2}q_{e}t}$$

reflects the trend of the adsorption processes that require a longer time to fill the adsorption sites.

**Figure 9:**
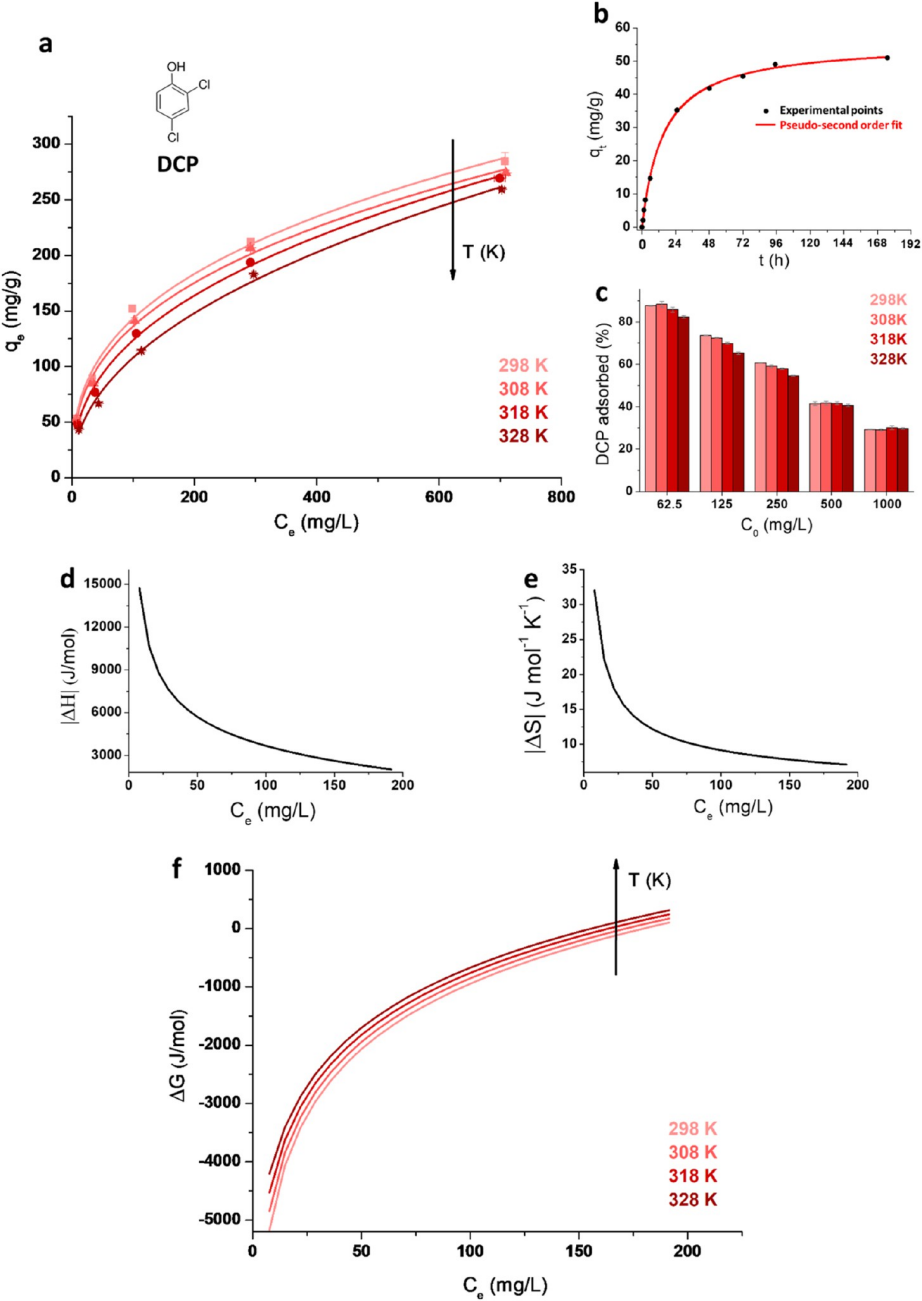
Experimental points (dots) and fitting curves of DCP equilibrium adsorption by xCNF-PVBC at 298, 308, 318, and 328 K (a) and adsorption kinetics of DCP from a 62.5 mg/L solution by xCNF-PVBC at 298 K (b); percentage of DCP adsorbed at 298, 308, 318, and 328 K by xCNF-PVBC versus the initial concentration *C*_0_ (c); thermodynamic parameters of DCP adsorption by xCNF-PVBC: enthalpy (d), entropy (e), and Gibbs free energy changes (f).

The results obtained for xCNF-PVBC (Figure 9b) fit well with both the PFO and PSO kinetic models in the first 24 h, while the whole curve is best fitted by the PSO kinetics (Table S1 and Figure S3). About 51 mg/g of DCP is adsorbed at equilibrium for xCNF-PVBC, of which about 69% is adsorbed in the first 24 h. Instead, the amount of DCP adsorbed on CNF (*q*_e_) is much lower, but the rate of adsorption is higher. Under the same conditions, only 10 mg/g of DCP is adsorbed, and equilibrium is reached in about 1 h. Also, in this case, the PFO and PSO models show a similar fit to the kinetic curve, with the PFO model providing a slightly better fit. The faster kinetics observed for CNF can be attributed to the fact that their interactions with DCP occur exclusively through hydrogen bonding with cellulose fibrils. In contrast, in the case of xCNF-PVBC, the adsorbent–adsorbate interactions are kinetically limited by the diffusion of DCP molecules into the micro/mesoporous structure. On the other hand, the equilibrium adsorption isotherms show that, when *C*_0_increases, the adsorption capacity of xCNF-PVBC progressively increases, while *q*_e_ of pristine CNF does not increase significantly.

Indeed, the equilibrium adsorption tests show that by increasing the pollutant concentration, the xCNF-PBVC adsorption capacity increases at all temperatures (Figure 9a). This behavior is typical of physisorption processes in micro/mesoporous materials, where adsorption also takes place in multilayers. Indeed, Freundlich and Langmuir models were employed to fit the data, and all curves show a better correlation with the Freundlich model (Table S2 in Supporting Information). The Freundlich model accounts for multilayer adsorption and expresses the relation between the equilibrium adsorption and the equilibrium concentration through the equation

4 $$q_{e}=K_{F}C_{e}^{1/\eta }$$

where *q*_e_ is the equilibrium adsorption, *C*_e_ is the equilibrium concentration, *K*_F_ is the Freundlich constant, which is indicative of the adsorption capacity, and η is an empirical constant related to the heterogeneity of the adsorbent surface. The higher the maximum capacity, the higher the Freundlich coefficient *K*_F_.^47,48^

The percentage of DCP adsorbed as a function of temperature and concentration changes is shown in Figure 9c. As shown, at the lowest concentration investigated, xCNF-PVBC adsorbed up to 87% DCP.

The uptake of DCP by xCNF-PVBC decreases with increasing temperature, demonstrating the exothermic nature of the process. The thermodynamic adsorption parameters were evaluated as follows. The Gibbs energy change (J/mol) is calculated using eq 3:

5 ΔG=−RT⁡ln⁡K

where the constant *K* can be expressed as

6 $$K=\frac{C_{\mathrm{ad}}}{C_{e}}$$

in which *C*_ad_ (mg/L) and *C*_e_ (mg/L) are the concentrations of solute adsorbed at equilibrium and the solute concentration in solution at equilibrium, respectively, *R* is the gas constant (=8.314 J/mol K), and *T* is the absolute temperature in kelvins. The relationship between the Gibbs energy change and the enthalpy change Δ*H* (J/mol) and entropy change Δ*S* (J/mol K) of adsorption is expressed as

7 ΔG=ΔH−TΔS

Substituting eq 3 into eq 5 gives

8 $$\ln K=-\frac{\Delta H}{\mathrm{RT}}+\frac{\Delta S}{R}$$

The values of Δ*H* and Δ*S* are determined from the slope and intercept of the linear plot of ln *K* versus 1/*T* of eq 8. *K* and ln *K* curves versus concentration were obtained by fitting the *C*_ad_ values for the adsorption isotherms. The results of Δ*G*, Δ*H*, and Δ*S* are shown in Figure 9d–f. For xCNF-PVBC, enthalpy and entropy changes are both negative contributions, as is usual in physical adsorption; in Figure 9d,e, they are shown in their absolute values.

As reported in Figure 9d, the absolute value of the enthalpy change decreases with increasing concentration. This is because at low concentrations, the monolayer is still forming, and the specific interactions of adsorbates with the surface of micro/mesopores (van der Waals interactions and π–π interactions) and with cellulose functional groups (hydrogen bonding) are still accessible. Higher concentrations, on the other hand, are related to the formation of multilayers in which direct contact with the pore surface and specific groups of cellulose is prevented. Under these conditions, the effect of the newly generated micro/mesoporosity is the driving force of adsorption. Briefly, xCNF-PVBC is able to adsorb DCP through specific interactions with the cellulose and PVBC functional groups and through van der Waals interactions in the micro/mesoporosity of the hyper-cross-linked shell of the cellulose nanofibrils (see also the scheme in Figure 10a).

**Figure 10:**
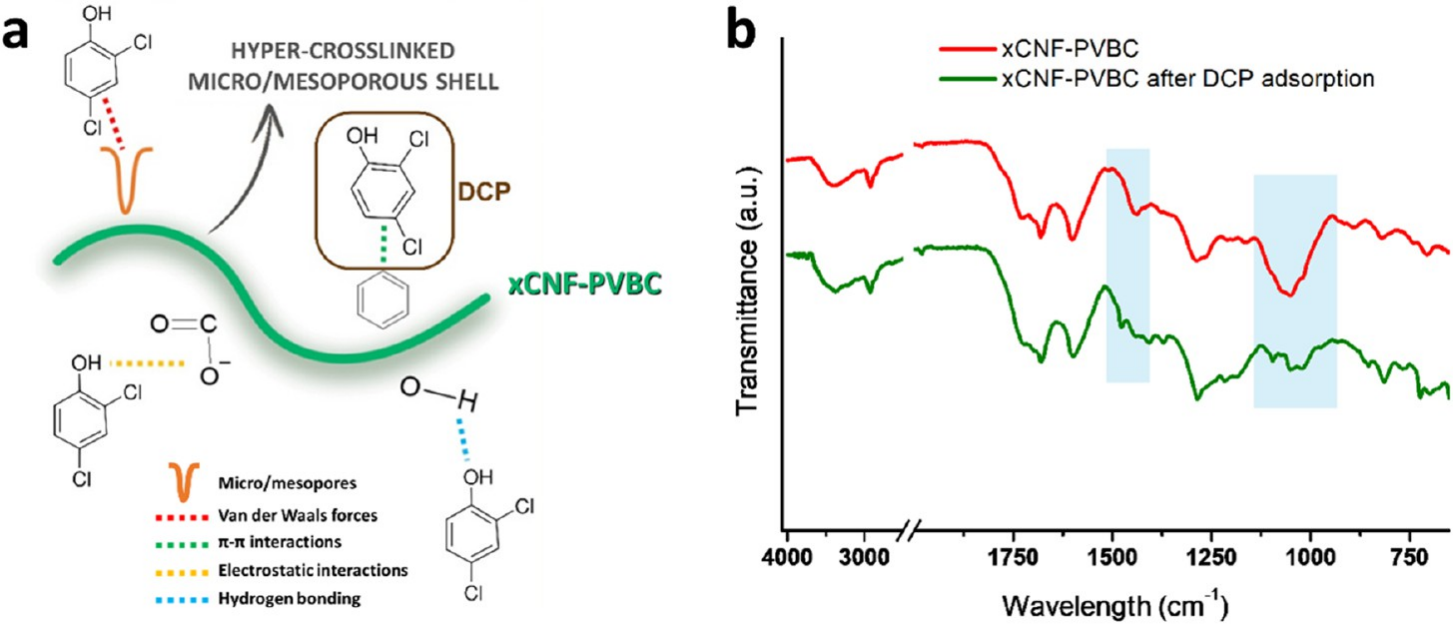
Schematic representation of possible xCNF-PVBC adsorption mechanisms (a) and FTIR spectra of xCNF-PVBC and xCNF-PVBC after DCP adsorption (b).

The π–π interactions can be confirmed by the blue shift of C=C stretching from 1441 to 1477 cm^–1^, while the spectrum variations in the 940–1140 cm^–1^ region, which is attributed to the stretching vibrations of glycosidic C–O bonds and the C–O bonds of alcohol groups, may be related to specific O–H interactions with DCP (Figure 10b).^49^

At higher concentrations of the pollutants, the entropy change Δ*S* decreases with increasing *C*_e_ (Figure 9e) since the system becomes increasingly ordered and the entropy difference due to the adsorption of a molecule, which is added in multilayer coverage, decreases. In Figure 9f, the Gibbs free energy is shown at different temperatures. The negative Gibbs free energy values indicate the feasibility and spontaneous nature of the adsorption process,^50^ which is more favored at lower temperatures since Gibbs free energy values are lower.^51^ The maximum enthalpy change value of xCNF-PVBC is −15 kJ/mol, and the maximum value of entropy change is −32 J/mol K. Therefore, the values of Δ*H* indicate that the adsorption phenomenon is purely physical since typical values for physisorption are between −20 and 0 kJ/mol.^52^ The negative values of Δ*H* confirm that the adsorption process of DCP by xCNF-PVBC is exothermic and occurs through physisorption. Similar values are obtained in the literature, for example, by Zhou et al. for the same pollutant.^52^

The adsorption properties of the xCNF-PVBC developed in this work were compared to those of other materials based on cellulose or hyper-cross-linked polymers, and tested for the adsorption of chlorophenols or other halogenated aromatic pollutants (Table 2). Compared to other cellulose-based materials, such as acid-modified plantain peel,^53^ highly cross-linked cellulose^54^ and copper-modified nanocellulose/alginate hybrid hydrogels,^55^ xCNF-PVBC exhibits significantly higher SSA and adsorption capacity for organic pollutants. On the other hand, comparing the adsorption capacity of xCNF-PVBC for fossil-based resins such as hyper-cross-linked 1*H*-benzotriazole,^56^ N-containing hyper-cross-linked polymers,^57^ cross-linked waste polystyrene,^58^ and phenyl-rich β-cyclodextrin cross-linked polymer,^59^ DCP uptake by xCNF-PVBC is quite high, considering the lower value of SSA of xCNF-PVBC compared to these fossil-based resins.

**Table 2:** Literature Adsorbents Based on Cellulose or Employed for the Adsorption of Chlorophenols Compared with xCNF-PVBC

adsorbent	SSA (m^2^/g)	adsorbate	*Q*_e_ (mg/g)	*C*_0_ (mg/L)	temperature (K)	ref
modified plantain peel as a cellulose-based adsorbent	32.3	2,6-DCP	16 ca.	500	303	^53^
copper-modified cellulose nanocrystal-based hydrogel spheres	50	4-CP	50 ca.	1000	303	^54^
hyper-cross-linked cellulose	148	niflumic acid	40	100	298	^55^
		paracetamol	30	100	298	^55^
N-heterocyclic hyper-cross-linked polymer	1023	4-CP	345.8	1000	303	^56^
N-containing hyper-cross-linked polymer	1544	2,4-DCP	350 ca.	65.2	295	^57^
cross-linked waste polystyrene	540	4-CP	268	500	303	^58^
phenyl-rich β-cyclodextrin porous cross-linked polymer	1099	2,4,6-TCP	700 ca.	200	298	^59^
	201	2,4,6-TCP	500 ca.	200	298	^59^
hyper-cross-linked poly(vinylbenzyl chloride)-functionalized cellulose nanofibrils (xCNF-PVBC)	409	2,4-DCP	284	1000	298	this work

In order to assess the contribution of cellulose nanofibrils to the adsorption of DCP in the functionalized sample xCNF-PVBC, the equilibrium adsorption of CNF was also tested at 298 and 328 K (Figure S2b). CNF reach plateau adsorption values in all cases in the investigated range, with a maximum uptake of about 13.5 mg/g at 328 K. Freundlich and Langmuir models were applied to the equilibrium adsorption isotherms of CNF and, although fitting correlations are not high in both cases due to the scattering of the experimental points, the Langmuir model is clearly more appropriate to describe CNF adsorption isotherms (Table S3). Therefore, the results show that the adsorption capacity of CNF slightly increases with temperature, indicating that the adsorption of DCP from water solution onto CNF is an endothermic process, in which increasing the temperature, intermolecular forces between DCP and CNF become stronger than those between DCP and water.

The recyclability of the functionalized cellulose nanofibrils xCNF-PVBC was evaluated through repeated adsorption/regeneration cycles (Figure 11a). The regeneration efficiency is above 98% over five repeated adsorption cycles, demonstrating the high desorption capacity of xCNF-PVBC and the possibility to reuse the adsorbent multiple times. DCP desorption was conducted by ethanol washes and promoted by moderate heating, further corroborating the physical character of the adsorption mechanism of xCNF-PVBC.

**Figure 11:**
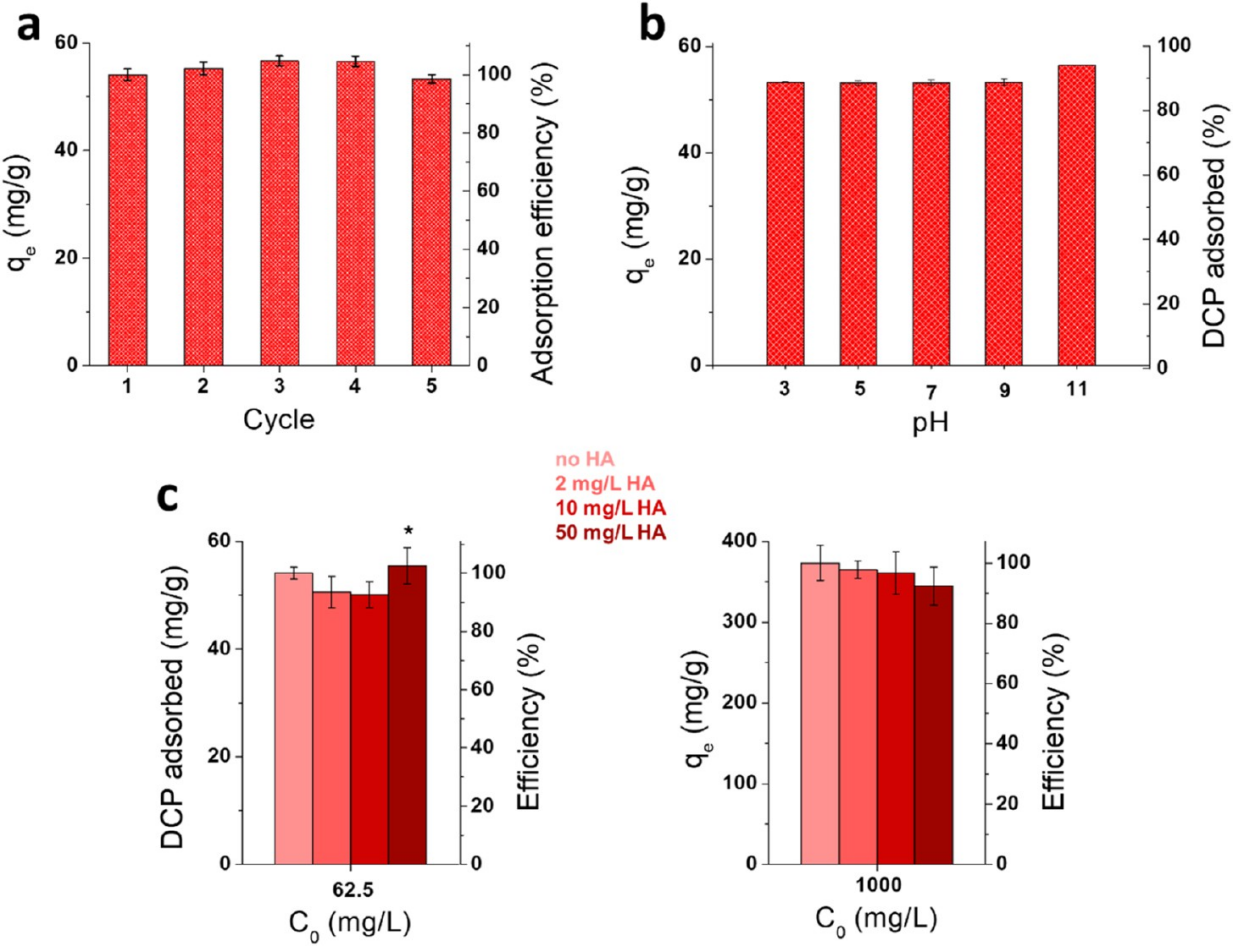
Adsorption efficiency of DCP (*C*_0_ = 62.5 mg/L) by xCNF-PVBC in repeated adsorption/regeneration cycles (a); effect of pH (b) and HA (c) on the adsorption of DCP.

The effect of the pH of the solution on DCP adsorption onto xCNF-PVBC was evaluated. As shown in Figure 11b, pH variation does not significantly affect DCP adsorption, apart from a slight increase observed at pH 11. Under alkaline conditions, DCP may undergo deprotonation, which could reduce its solubility and thereby enhance hydrophobic interactions with the adsorbent surface.^60^ The potential interference of coexisting organic contaminants with the adsorption of DCP by xCNF-PVBC was evaluated. Aquatic environments typically contain natural organic matter, with humic acids being its major component.^61–64^ The capacity of adsorption of DCP by xCNF-PVBC in the presence of HA was evaluated at the lowest and highest concentrations considered in previous experiments (62.5 and 1000 mg/L) and at variable HA concentrations from 2 to 50 mg/L. As shown in Figure 11b, the presence of HA does not significantly affect the adsorption of DCP by xCNF-PVBC, with the efficiency of adsorption being above 90% in all cases. In the tests performed with DCP at a higher initial concentration (1000 mg/L), the efficiency is about 98 and 97% at 2 mg/L and 10 mg/L HA, respectively, and decreases to 92% with increasing HA concentration at 50 mg/L, as expected. In the tests performed with a lower DCP initial concentration (i.e., 62.5 mg/L), the presence of HA, inducing a slight increase of the UV spectra baseline, causes moderate detection issues of the concentration of DCP, resulting in scattering results, especially at the highest HA concentration (i.e., 50 mg/L). However, in all cases, the adsorption of DCP in the presence of HA is reduced by less than 10% with respect to the values shown in the tests without HA, demonstrating moderate competitive adsorption between the target pollutant and the model natural organic matter.

## 4 Conclusions

In this work, a novel high surface area adsorbent based on cellulose and inspired by hyper-cross-linked polymers was designed. Cellulose nanofibrils were functionalized with poly­(vinylbenzyl chloride) and subjected to Friedel–Crafts alkylation, generating a micro/mesoporous adsorbent (xCNF-PVBC) characterized by an SSA of 409 m^2^/g and a microporous fraction of 50%. xCNF-PVBC exhibited excellent performance in the removal of DCP, a persistent aromatic and halogenated pollutant, achieving up to 90% removal at 62.5 mg/L and an adsorption capacity of 284 mg/g at higher concentrations (1000 mg/L). Thermodynamic analysis confirmed that the adsorption process was energetically favorable, and the material demonstrated outstanding reusability, maintaining over 98% efficiency across 5 adsorption/desorption cycles. Furthermore, the adsorption performance was stable across varying pH levels, and interference from natural organic matter (e.g., humic acids) was minimal (<10%).

While the material is not yet fully biobased, it currently consists of approximately 70 wt % renewable content. This represents a significant step toward more sustainable sorbent technologies and highlights the feasibility of utilizing cellulose nanofibrils as functional scaffolds for the development of competitive alternatives to conventional fossil-based adsorbents.

Importantly, this approach opens new avenues for future development. This strategy may be extended to cellulose-rich feedstocks derived from agro-industrial residues or agricultural waste, promoting circular economic principles. Moreover, replacing the fossil-derived aromatic polymer phase (PVBC) with biobased aromatic compounds such as polyphenols could enable the creation of fully biobased and sustainable porous adsorbents.

## Supplementary Material



## Notes

^13^C CPMAS NMR spectrum of CNF_24h, adsorption tests of DCP by CNF, kinetics parameters and curves of DCP adsorption, and the Freundlich and Langmuir fitting parameters of DCP adsorption isotherms (PDF)
