# Supplementary material for: Hyper-Cross-linked Cellulose Nanofibrils with Spontaneous and Reversible Adsorption of Aromatic Pollutants from Water as a Valid Alternative to Fossil-Based Adsorbents
Source: ACS Appl Mater Interfaces. 2025 Jun 20;17(26):38619–34. doi: 10.1021/acsami.5c05009 (PMC12232286; doi:10.1021/acsami.5c05009)
Supplement: Supplementary file 1 [file am5c05009_si_001.pdf]

Hyper-crosslinked cellulose nanofibrils with spontaneous and reversible adsorption of aromatic pollutants from water as a valid alternative to fossil-based adsorbents

Antonio Maglione[1], Federico Olivieri[1], Roberto Avolio[1], Rachele Castaldo[1*], Mariacristina Cocca[1], Maria Emanuela Errico[1], Veronica Ambrogi[2], Gennaro Gentile[1]

[1] Institute for Polymers Composites and Biomaterials, National Research Council of Italy, Via Campi Flegrei 34, 80078 Pozzuoli, Italy

[2] Department of Chemical, Materials and Industrial Production Engineering, University of Naples Federico II, P.le Tecchio 80, 80125 Napoli, Italy

Supplementary information

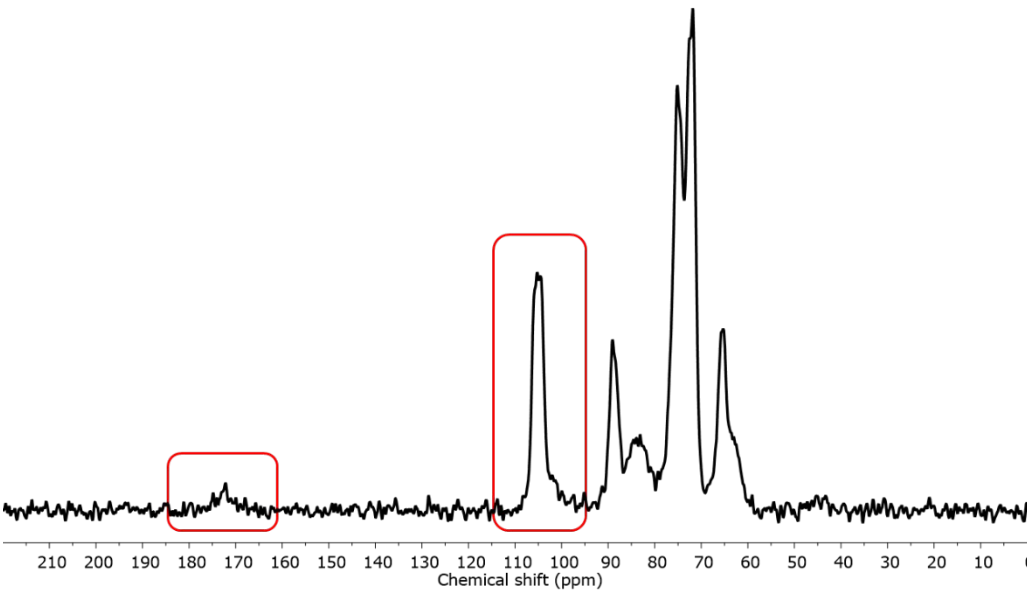

Figure S1. ^{13}C CPMAS NMR spectrum of CNF_24h.

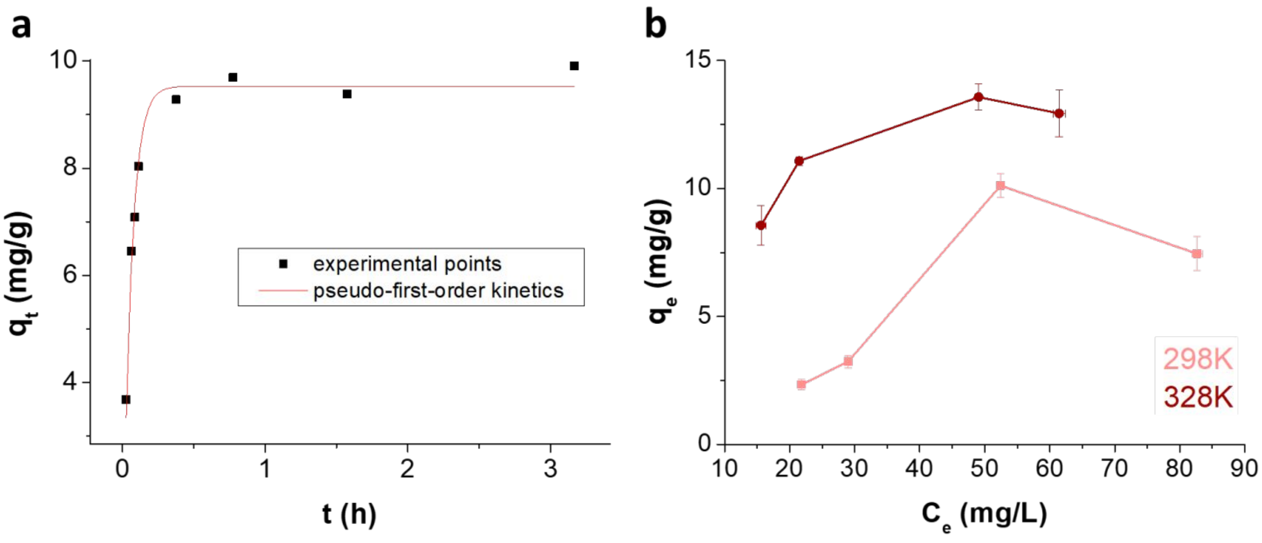

Figure S2. Adsorption tests of DCP by CNF: kinetic test from a 62.5 mg/L solution at 298 K (a) and equilibrium tests at 298 K and 328 K (b).

Table S1. Pseudo-first-order and pseudo-second-order kinetics parameters of DCP adsorption (62.5 mg/L) onto xCNF-PVBC and CNF at 298 K.

	Pseudo-first-order			Pseudo-second-order		
	q_e (mg/g)	k_1 (h^{-1})	R^2	q_e (mg/g)	k_2 (min^{-1})	R^2
xCNF-PVBC	48.3 ± 1.2	0.05 ± 0.0058	0.991	55.7 ± 0.65	1.19E-3 ± 7.02E-5	0.999
CNF	9.53 ± 0.15	17.27 ± 1.01	0.981	10.03 ± 0.18	2.76 ± 0.30	0.978

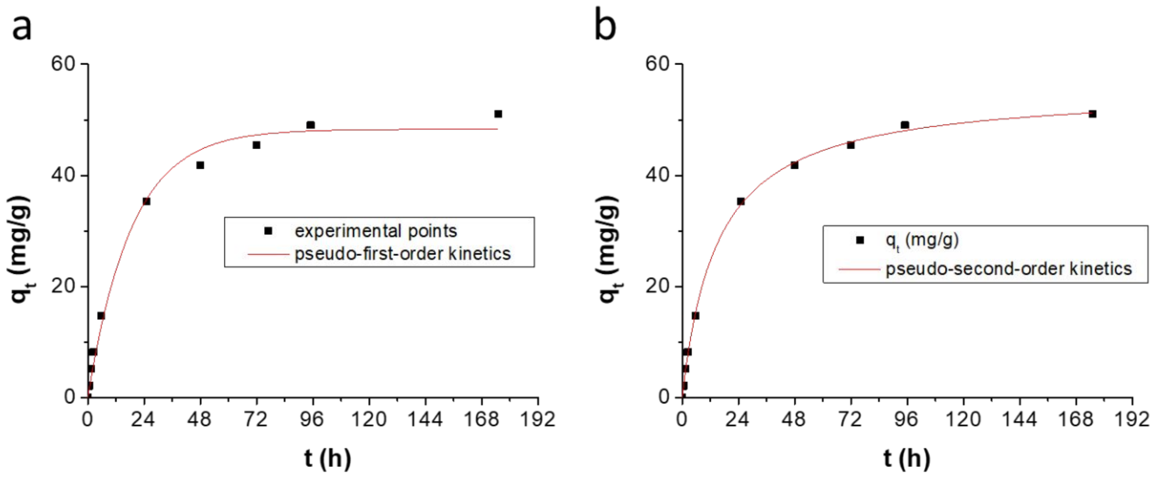

Figure S3. PFO (a) and PSO (b) kinetics models fit of DCP adsorption (62.5 mg/L) onto xCNF-PVBC at 298 K.

Table S2. Freundlich and Langmuir fitting parameters for xCNF-PVBC equilibrium DCP adsorption isotherms.

Temperature	Freundlich			Langmuir		
	n	K_F	R^2	q_m (mg/g)	K_L (L/mg)	R^2
298 K	2.66	1.04	0.995	197	0.046	0.926
308 K	2.71	1.00	0.995	180	0.054	0.893
318 K	2.48	0.92	0.995	174	0.041	0.896
328 K	2.25	0.83	0.991	162	0.032	0.883

Table S3. Freundlich and Langmuir fitting parameters for CNF equilibrium DCP adsorption isotherms.

Temperature	Freundlich			Langmuir		
	n	K_F	R^2	q_m (mg/g)	K_L (L/mg)	R^2
298 K	0.99	0.11	0.725	-165	-0.001	0.861
328 K	3.43	0.11	0.844	17	0.072	0.909
